# Decreased Self-reported Physical Fitness Following SARS-CoV-2 Infection and the Impact of Vaccine Boosters in a Cohort Study

**DOI:** 10.1093/ofid/ofad579

**Published:** 2023-11-17

**Authors:** Stephanie A Richard, Ann I Scher, Jennifer Rusiecki, Celia Byrne, Catherine M Berjohn, Anthony C Fries, Tahaniyat Lalani, Alfred G Smith, Rupal M Mody, Anuradha Ganesan, Nikhil Huprikar, Rhonda E Colombo, Christopher J Colombo, Christina Schofield, David A Lindholm, Katrin Mende, Michael J Morris, Milissa U Jones, Ryan Flanagan, Derek T Larson, Evan C Ewers, Samantha E Bazan, David Saunders, Ryan C Maves, Jeffrey Livezey, Carlos J Maldonado, Margaret Sanchez Edwards, Julia S Rozman, Robert J O’Connell, Mark P Simons, David R Tribble, Brian K Agan, Timothy H Burgess, Simon D Pollett, the EPICC COVID-19 Cohort Study Group, Col J Cowden, LTC M Darling, S DeLeon, Maj D Lindholm, LTC A Markelz, K Mende, S Merritt, T Merritt, LTC N Turner, CPT T Wellington, Carl R, LTC S Bazan, P K Love, Alexander T, N Dimascio-Johnson, N Elnahas, MAJ E Ewers, LCDR K Gallagher, C Glinn, U Jarral, D Jennings, LCDR D Larson, K Reterstoff, A Rutt, A Silva, C West, Henry M, P Blair, J Chenoweth, D Clark, J Bowman, S Chambers, LTC C Colombo, R Colombo, CPT C Conlon, CPT K Everson, COL P Faestel, COL T Ferguson, MAJ L Gordon, LTC S Grogan, CPT S Lis, M Martin, COL C Mount, LTC D Musfeldt, CPT D Odineal, LTC M Perreault, W Robb-McGrath, MAJ R Sainato, C Schofield, COL C Skinner, M Stein, MAJ M Switzer, MAJ M Timlin, MAJ S Wood, S Banks, R Carpenter, L Kim, CAPT K Kronmann, T Lalani, LCDR T Lee, LCDR A Smith, R Smith, R Tant, CDR T Warkentien, CDR C Berjohn, S Cammarata, N Kirkland, D Libraty, R Maves, G Utz, C Bradley, S Chi, LTC R Flanagan, A Fuentes, MAJ M Jones, N Leslie, C Lucas, C Madar, K Miyasato, C Uyehara, H Adams, B Agan, L Andronescu, A Austin, C Broder, CAPT T Burgess, C Byrne, K Chung, J Davies, C English, N Epsi, C Fox, M Fritschlanski, A Hadley, COL P Hickey, E Laing, LTC C Lanteri, LTC J Livezey, A Malloy, R Mohammed, C Morales, P Nwachukwu, C Olsen, E Parmelee, S Pollett, S Richard, J Rozman, J Rusiecki, COL D Saunders, E Samuels, M Sanchez, A Scher, CDR M Simons, A Snow, K Telu, D Tribble, M Tso, L Ulomi, M Wayman, N Hockenbury, TSgt T Chao, R Chapleau, M Christian, A Fries, C Harrington, V Hogan, S Huntsberger, K Lanter, E Macias, J Meyer, S Purves, K Reynolds, J Rodriguez, C Starr, CAPT J Iskander, CDR I Kamara, B Barton, LTC D Hostler, LTC J Hostler, MAJ K Lago, C Maldonado, J Mehrer, MAJ T Hunter, J Mejia, R Mody, J Montes, R Resendez, P Sandoval, I Barahona, A Baya, A Ganesan, MAJ N Huprikar, B Johnson, S Peel

**Affiliations:** Infectious Disease Clinical Research Program, Department of Preventive Medicine and Biostatistics, Uniformed Services University of the Health Sciences, Bethesda, Maryland, USA; Henry M. Jackson Foundation for the Advancement of Military Medicine, Inc., Bethesda, Maryland, USA; Uniformed Services University of the Health Sciences, Bethesda, Maryland, USA; Uniformed Services University of the Health Sciences, Bethesda, Maryland, USA; Uniformed Services University of the Health Sciences, Bethesda, Maryland, USA; Infectious Disease Clinical Research Program, Department of Preventive Medicine and Biostatistics, Uniformed Services University of the Health Sciences, Bethesda, Maryland, USA; Uniformed Services University of the Health Sciences, Bethesda, Maryland, USA; Naval Medical Center San Diego, San Diego, California, USA; US Air Force School of Aerospace Medicine, Wright-Patterson, Ohio, USA; Infectious Disease Clinical Research Program, Department of Preventive Medicine and Biostatistics, Uniformed Services University of the Health Sciences, Bethesda, Maryland, USA; Henry M. Jackson Foundation for the Advancement of Military Medicine, Inc., Bethesda, Maryland, USA; Naval Medical Center Portsmouth, Portsmouth, Virginia, USA; Naval Medical Center Portsmouth, Portsmouth, Virginia, USA; William Beaumont Army Medical Center, El Paso, Texas, USA; Infectious Disease Clinical Research Program, Department of Preventive Medicine and Biostatistics, Uniformed Services University of the Health Sciences, Bethesda, Maryland, USA; Henry M. Jackson Foundation for the Advancement of Military Medicine, Inc., Bethesda, Maryland, USA; Walter Reed National Military Medical Center, Bethesda, Maryland, USA; Uniformed Services University of the Health Sciences, Bethesda, Maryland, USA; Walter Reed National Military Medical Center, Bethesda, Maryland, USA; Infectious Disease Clinical Research Program, Department of Preventive Medicine and Biostatistics, Uniformed Services University of the Health Sciences, Bethesda, Maryland, USA; Henry M. Jackson Foundation for the Advancement of Military Medicine, Inc., Bethesda, Maryland, USA; Uniformed Services University of the Health Sciences, Bethesda, Maryland, USA; Madigan Army Medical Center, Joint Base Lewis McChord, Washington, USA; Uniformed Services University of the Health Sciences, Bethesda, Maryland, USA; Madigan Army Medical Center, Joint Base Lewis McChord, Washington, USA; Madigan Army Medical Center, Joint Base Lewis McChord, Washington, USA; Uniformed Services University of the Health Sciences, Bethesda, Maryland, USA; Brooke Army Medical Center, Joint Base San Antonio-Fort Sam Houston, Texas, USA; Infectious Disease Clinical Research Program, Department of Preventive Medicine and Biostatistics, Uniformed Services University of the Health Sciences, Bethesda, Maryland, USA; Henry M. Jackson Foundation for the Advancement of Military Medicine, Inc., Bethesda, Maryland, USA; Brooke Army Medical Center, Joint Base San Antonio-Fort Sam Houston, Texas, USA; Brooke Army Medical Center, Joint Base San Antonio-Fort Sam Houston, Texas, USA; Department of Pediatrics, Translational Medicine Unit, Uniformed Services University, Bethesda, Maryland, USA; Department of Pediatrics, Translational Medicine Unit, Uniformed Services University, Bethesda, Maryland, USA; Uniformed Services University of the Health Sciences, Bethesda, Maryland, USA; Naval Medical Center San Diego, San Diego, California, USA; Alexander T. Augusta Military Medical Center, Fort Belvoir, Virginia, USA; Uniformed Services University of the Health Sciences, Bethesda, Maryland, USA; Alexander T. Augusta Military Medical Center, Fort Belvoir, Virginia, USA; Carl R. Darnall Army Medical Center, Fort Cavazos, Texas, USA; Uniformed Services University of the Health Sciences, Bethesda, Maryland, USA; Wake Forest University School of Medicine, Winston-Salem, North Carolina, USA; Department of Pediatrics, Clinical Pharmacology and Medical Toxicology, Uniformed Services University of the Health Sciences, Bethesda, Maryland, USA; Womack Army Medical Center, Fort Liberty, North Carolina, USA; Infectious Disease Clinical Research Program, Department of Preventive Medicine and Biostatistics, Uniformed Services University of the Health Sciences, Bethesda, Maryland, USA; Henry M. Jackson Foundation for the Advancement of Military Medicine, Inc., Bethesda, Maryland, USA; Infectious Disease Clinical Research Program, Department of Preventive Medicine and Biostatistics, Uniformed Services University of the Health Sciences, Bethesda, Maryland, USA; Henry M. Jackson Foundation for the Advancement of Military Medicine, Inc., Bethesda, Maryland, USA; Infectious Disease Clinical Research Program, Department of Preventive Medicine and Biostatistics, Uniformed Services University of the Health Sciences, Bethesda, Maryland, USA; Infectious Disease Clinical Research Program, Department of Preventive Medicine and Biostatistics, Uniformed Services University of the Health Sciences, Bethesda, Maryland, USA; Infectious Disease Clinical Research Program, Department of Preventive Medicine and Biostatistics, Uniformed Services University of the Health Sciences, Bethesda, Maryland, USA; Infectious Disease Clinical Research Program, Department of Preventive Medicine and Biostatistics, Uniformed Services University of the Health Sciences, Bethesda, Maryland, USA; Henry M. Jackson Foundation for the Advancement of Military Medicine, Inc., Bethesda, Maryland, USA; Infectious Disease Clinical Research Program, Department of Preventive Medicine and Biostatistics, Uniformed Services University of the Health Sciences, Bethesda, Maryland, USA; Infectious Disease Clinical Research Program, Department of Preventive Medicine and Biostatistics, Uniformed Services University of the Health Sciences, Bethesda, Maryland, USA; Henry M. Jackson Foundation for the Advancement of Military Medicine, Inc., Bethesda, Maryland, USA

**Keywords:** COVID-19, fitness, long COVID

## Abstract

**Background:**

The long-term effects of coronavirus disease 2019 (COVID-19) on physical fitness are unclear, and the impact of vaccination on that relationship is uncertain.

**Methods:**

We compared survey responses in a 1-year study of US military service members with (n = 1923) and without (n = 1591) a history of severe acute respiratory syndrome coronavirus 2 (SARS-CoV-2) infection. We fit Poisson regression models to estimate the association between history of SARS-CoV-2 infection and fitness impairment, adjusting for time since infection, demographics, and baseline health.

**Results:**

The participants in this analysis were primarily young adults aged 18–39 years (75%), and 71.5% were male. Participants with a history of SARS-CoV-2 infection were more likely to report difficulty exercising (38.7% vs 18.4%; *P* < .01), difficulty performing daily activities (30.4% vs 12.7%; *P* < .01), and decreased fitness test (FT) scores (42.7% vs 26.2%; *P* < .01) than those without a history of infection. SARS-CoV-2-infected participants were at higher risk of these outcomes after adjusting for other factors (unvaccinated: exercising: adjusted risk ratio [aRR], 3.99; 95% CI, 3.36–4.73; activities: aRR, 5.02; 95% CI, 4.09–6.16; FT affected: aRR, 2.55; 95% CI, 2.19–2.98). Among SARS-CoV-2-positive participants, full vaccination before infection was associated with a lower risk of post-COVID-19 fitness impairment (fully vaccinated: exercise: aRR, 0.81; 95% CI, 0.70–0.95; activities: aRR, 0.76; 95% CI, 0.64–0.91; FT: aRR, 0.87; 95% CI, 0.76–1.00; boosted: exercise: aRR, 0.62; 95% CI, 0.51–0.74; activities: aRR, 0.52; 95% CI, 0.41–0.65; FT: aRR, 0.59; 95% CI, 0.49–0.70).

**Conclusions:**

In this study of generally young, healthy military service members, SARS-CoV-2 infection was associated with lower self-reported fitness and exercise capacity; vaccination and boosting were associated with lower risk of self-reported fitness loss.

There is growing evidence to support a causal link between coronavirus disease 2019 (COVID-19) and impaired cardiorespiratory health, particularly in those with severe COVID-19, older age, and preexisting comorbidities [1]. Multiple studies have shown that such postacute sequelae include loss of fitness as measured by cardiopulmonary exercise testing, but older age groups and those with severe COVID-19 requiring hospitalization were generally overrepresented in these studies [2]. In such groups, the described post-hospitalization fitness loss may be nonspecific and reflect expected deconditioning, which is common after hospitalization for other acute conditions, particularly in older adults [3]. By comparison, there are few data on the fitness impact in younger age groups with milder acute COVID-19.

Moreover, it is unclear whether COVID-19 vaccination and vaccine boosting mitigate any risk of post-COVID fitness impairment, including in those who only experience mild acute COVID-19 [4]. Understanding whether COVID-19 booster receipt may mitigate functional fitness loss is particularly important as the majority of younger populations remain unboosted and a substantial proportion remain unvaccinated [5]. While there is emerging evidence that COVID-19 vaccination mitigates the risk of postacute symptoms [6–9], other studies have found no effect on post-COVID conditions (“long COVID”) [10, 11]. It is unknown how effective the vaccine primary series and further boosting may be in preventing fitness loss, required for quality of life, employment, and key societal roles.

Addressing this issue is important for professions requiring optimal fitness such as the military and other hazardous and physically demanding professions [3]. Military populations, in turn, afford a unique opportunity to answer this question, as they require regular service-mandated fitness testing (FT) and are generally at low risk for severe acute COVID-19 due to low rates of comorbidities. Military service members are required to pass a physical fitness test on a regular basis (specific test and frequency depend on military branch) that assesses strength and cardiovascular fitness. Multiple studies have now characterized COVID-19 in US Military Health System (MHS) beneficiaries [12], including active duty service members, describing their hospitalization risk as well as the burden of acute and persistent symptoms [13–16] and post-COVID medical care [9]. Current evidence that COVID-19 may limit the physical fitness in a military population is limited. For example, Crameri et al. noted reduced predicted maximal aerobic capacity in Swiss military recruits 1–2 months after symptomatic COVID-19 [17], and O'Sullivan et al. found that British military personnel who had been hospitalized for COVID-19 were more likely to have poor cardiopulmonary function at 5 months postinfection than those who were not infected with SARS-CoV-2 [18].

COVID-19 vaccination (primary series) was mandatory for US service members from August 2021 through December 2022. Vaccine boosting remains optional (though recommended) for military personnel, and currently uptake is low [19]. Evaluating whether vaccination and boosting may protect against post-COVID fitness decrements may inform vaccination recommendations for military populations as well as other groups, including those at low risk for severe COVID-19 and in whom booster uptake has been similarly low [20]. Aside from vaccination history, identification of other specific risk factors for fitness impairment may assist in risk assessment as well as targeted interventions aimed at reducing post-COVID conditions [17].

We sought to describe the relationship between history of severe acute respiratory syndrome coronavirus 2 (SARS-CoV-2) infection and several measures of US military service members’ fitness (difficulty exercising, difficulty with daily activities, and decreased FT scores) among participants enrolled in the Epidemiology, Immunology, and Clinical Characteristics of Emerging Infectious Diseases with Pandemic Potential (EPICC) study. We extended this analysis to identify the duration of impaired physical fitness symptoms among those with a history of COVID-19, compared with those without a known history of COVID-19, hypothesizing that individuals with a history of SARS-CoV-2 infection were more likely to report impaired physical fitness and to have longer duration of impaired physical fitness than those without a known history of SARS-CoV-2 infection. Finally, we examined factors potentially associated with self-reported fitness changes, particularly vaccination and booster history, in addition to body mass index (BMI) and demographic characteristics.

## METHODS

### Patient Consent

The EPICC study was approved by the Uniformed Services University Institutional Review Board (IDCRP-085), and all study participants provided written consent when enrolled in the study. This observational cohort study in a convenience sample of MHS beneficiaries was conducted following good clinical practice and according to the Declaration of Helsinki guidelines.

### Study Population and Overall Cohort Description

The EPICC study is a longitudinal cohort study that aims to describe the clinical and functional outcomes of SARS-CoV-2 infection in MHS beneficiaries (including active duty service members, dependents [spouses, children], and military retirees). The design of this study has been described previously [13, 21]. Eligible enrollees in EPICC included MHS beneficiary populations (including adults and children) with a history of confirmed COVID-19, COVID-like illness, or exposure to SARS-CoV-2, those tested for SARS-CoV-2, and COVID-19 vaccine recipients. Participants were enrolled between March 2020 and May 2022 across 10 EPICC study sites and via an online recruitment pathway.

### Study Procedures, Including Measurement of Self-reported Fitness

Study procedures are summarized in Supplementary Table 1 and have been previously described [13, 21]. Demographic information and acute illness characteristics were collected using surveys and case report forms. Follow-up surveys and specimens were collected over 1 year (Supplementary Table 1). The Charlson Comorbidity Index (CCI) [22] was calculated using medical encounters in the MHS Data Repository (MDR) during the year before infection (those with a history of SARS-CoV-2 infection) or enrollment (those with no history of SARS-CoV-2 infection). The enrollment survey included a question about the participant's height and weight. BMI was calculated and categorized as normal (<25 kg/m^2^), overweight (25–29 kg/m^2^), obese (30–34 kg/m^2^), and severely obese (35+ kg/m^2^). COVID-19 vaccine history was ascertained both using surveys and through the participants’ medical histories in the MDR.

COVID-19 diagnosis was made from clinical polymerase chain reaction (PCR) results, participant report of positive respiratory swab test, or quantitative PCR (qPCR) on research swabs. As a large proportion of participants were enrolled via an online pathway without swab genotyping, we inferred infecting variant by a combination of infection date [23] and variant genotyping results performed on SARS-CoV-2-positive specimens. We categorized the study time periods as pre-Delta (2/28/20–6/14/21), Delta (6/15/21–12/31/21), and Omicron (1/1/22–5/31/22), estimated using the trends in the EPICC-derived SARS-CoV-2 sequence data and US data from the Global Initiative on Sharing Avian Influenza Data (GISAID). Hospitalization for COVID-19 was identified using surveys and medical record review. Participants were considered to be fully vaccinated if they had ≥2 doses of an mRNA vaccine (mRNA-1273/Moderna or BNT162b2/Pfizer-BioNTech) or ≥1 dose of JNJ78436735/Janssen ≥14 days before their first positive SARS-CoV-2 test. Participants who were partially vaccinated when they were infected (n = 37) were included as unvaccinated in the analyses. A participant was considered to be boosted if they had ≥1 vaccination received after full vaccination and that dose was given ≥14 days before their first positive SARS-CoV-2 test.

Subjects answered questions about whether they had new or increased difficulty exercising or doing daily activities (like walking or going up stairs) or if they felt that their FT score had been affected via electronic surveys implemented at enrollment and at 1, 3, 6, 9, and 12 months after enrollment. These survey questions were added in March 2021 and are detailed in Supplementary Table 2. Participants’ survey responses were categorized according to when the surveys were filled out relative to the first positive SARS-CoV-2 test date (within 2 weeks of the first positive test date [14 days before to 14 days after first positive test], 1 month [15 to 44 days after first positive test], 3 months [45 to 134 days], 6 months [135 to 224 days], 9 months [225 to 314 days], and 12 months [315 to 405 days]). Surveys that were completed >2 weeks before the first SARS-CoV-2-positive test date were categorized as “pre-SARS-CoV-2+,” and those who never tested positive were categorized as “never positive.” Those who responded that they had difficulty with exercising or daily activities were asked if the difficulty was due to fatigue, shortness of breath, joint pain, difficulty moving, or other (and they could choose as many as applied). The responses for each participant were summarized for each outcome in each timeframe and overall.

### Statistical Analysis

In this analysis, we included participants who were service members, with complete key characteristics (age, sex, BMI, CCI), ≥1 SARS-CoV-2 test, no known reinfections (defined as documented repeat positive SARS-CoV-2 tests >90 days apart), and who completed ≥1 follow-up survey that included the fitness questions. The 3 primary outcomes of this analysis were difficulty exercising, difficulty with daily activities, and reporting decreased FT scores.

We compared characteristics between those with and without a history of SARS-CoV-2 infection using Kruskal-Wallis rank-sum tests and Pearson chi-square tests, as appropriate. Multivariable Poisson regression models were fit separately for the outcomes of reporting difficulty exercising, difficulty with daily activities, and that their physical fitness test had been affected. Models controlled for time since first SARS-CoV-2-positive test (or enrollment for those who were never SARS-CoV-2 positive), their SARS-CoV-2 status (negative, unvaccinated positive, fully vaccinated positive), sex, age group (18–29, 30–39, 40+ years of age), BMI category, CCI [22] score >0, and military service affiliation, as well as a random effect for participant. Additional models were fit only in the participants who tested positive to identify the characteristics (including boosting status) associated with reporting these outcomes. For the analysis in the positive participants, participants were categorized as unvaccinated, fully vaccinated, or boosted at the time of infection. All analyses were run in R, version 4.1.2 (R Core Team [2017]). This report follows the STROBE reporting guidelines for cohort studies.

## RESULTS

Among the 7912 participants enrolled in EPICC, 5461 were service members, and 3514 met the inclusion criteria for the analysis (Supplementary Figure 1). The primary reasons for exclusion were missing surveys (n = 888), reinfections (n = 543), and missing BMI (n = 411). Among the participants included in this analysis, 92.4% (3247) had 12-month surveys. Participants were mostly young, male, and White (Table 1). The majority (3211, 91.4%) had a CCI of 0, and 880 (25.0%) had a BMI of ≥30. Hospitalization for COVID-19 was infrequent in this population (51, 2.7% of those with COVID-19). Among participants who had a history of SARS-CoV-2 infection (1923/3514, 54.7%), similar proportions were unvaccinated/partially vaccinated and recipients of the primary series (37.5% and 37.8%, respectively); a smaller percentage had received a booster before infection (24.7%).

**Table 1.** Characteristics of Active Duty US Military Health System Beneficiaries who Responded to ≥1 EPICC Survey With Fitness Questions

	SARS-CoV-2-(n = 1591), No. (%)	SARS-CoV-2+ (n = 1923), No. (%)	Total (n = 3514), No. (%)	*P* Value
Age group				.04^a^
18–29 y	559 (35.1)	598 (31.1)	1157 (32.9)	
30–39 y	646 (40.6)	828 (43.1)	1474 (41.9)	
40+ y	386 (24.3)	497 (25.8)	883 (25.1)	
Female	451 (28.3)	552 (28.7)	1003 (28.5)	.81^a^
Race/ethnicity				.17^a^
Asian	88 (5.5)	87 (4.5)	175 (5.0)	
Black	125 (7.9)	134 (7.0)	259 (7.4)	
Hispanic or Latino	224 (14.1)	311 (16.2)	535 (15.2)	
Other	173 (10.9)	187 (9.7)	360 (10.2)	
White	981 (61.7)	1204 (62.6)	2185 (62.2)	
Service branch				.02^a^
Air Force	301 (18.9)	363 (18.9)	664 (18.9)	
Army	549 (34.5)	728 (37.9)	1277 (36.3)	
Marines	83 (5.2)	116 (6.0)	199 (5.7)	
Navy	318 (20.0)	386 (20.1)	704 (20.0)	
Other	340 (21.4)	330 (17.2)	670 (19.1)	
BMI category				<.01^a^
Under/normal weight (BMI <25 kg/m^2^)	418 (26.3)	444 (23.1)	862 (24.5)	
Overweight (BMI 25–29 kg/m^2^)	825 (51.9)	947 (49.2)	1772 (50.4)	
Obese (BMI 30–34 kg/m^2^)	289 (18.2)	426 (22.2)	715 (20.3)	
Severely obese (BMI ≥35 kg/m^2^)	59 (3.7)	106 (5.5)	165 (4.7)	
Charlson comorbidity index category				.74^a^
0	1453 (91.3)	1758 (91.4)	3211 (91.4)	
1–2	129 (8.1)	151 (7.9)	280 (8.0)	
3–4	4 (0.3)	9 (0.5)	13 (0.4)	
5+	5 (0.3)	5 (0.3)	10 (0.3)	
Outpatient	1589 (99.9)	1872 (97.3)	3461 (98.5)	<.01^a^
Maximum days from first SARS-CoV-2 positive to survey				
Median (Q1, Q3)	NA	276.0 (136.0, 354.0)	276.0 (136.0, 354.0)	
Min–max	NA	15.0–404.0	15.0–404.0	
Vaccination status at time of SARS-CoV-2 infection				
Unvaccinated	NA	721 (37.5)	721 (37.5)	
Fully vaccinated	NA	727 (37.8)	727 (37.8)	
Boosted	NA	475 (24.7)	475 (24.7)	
Infected or enrolled during different variant periods				<.01^a^
Pre-Delta (2/28/20–6/14/21)	665 (41.8)	645 (33.5)	1310 (37.3)	
Delta (6/15/21–12/31/21)	734 (46.1)	425 (22.1)	1159 (33.0)	
Omicron (1/1/22+)	192 (12.1)	853 (44.4)	1045 (0.7)	

Abbreviations: BMI, body mass index; EPICC, Epidemiology, Immunology, and Clinical Characteristics of Emerging Infectious Diseases with Pandemic Potential; SARS-CoV-2, severe acute respiratory syndrome coronavirus 2.

^a^Pearson chi-square test.

**Figure 1.**
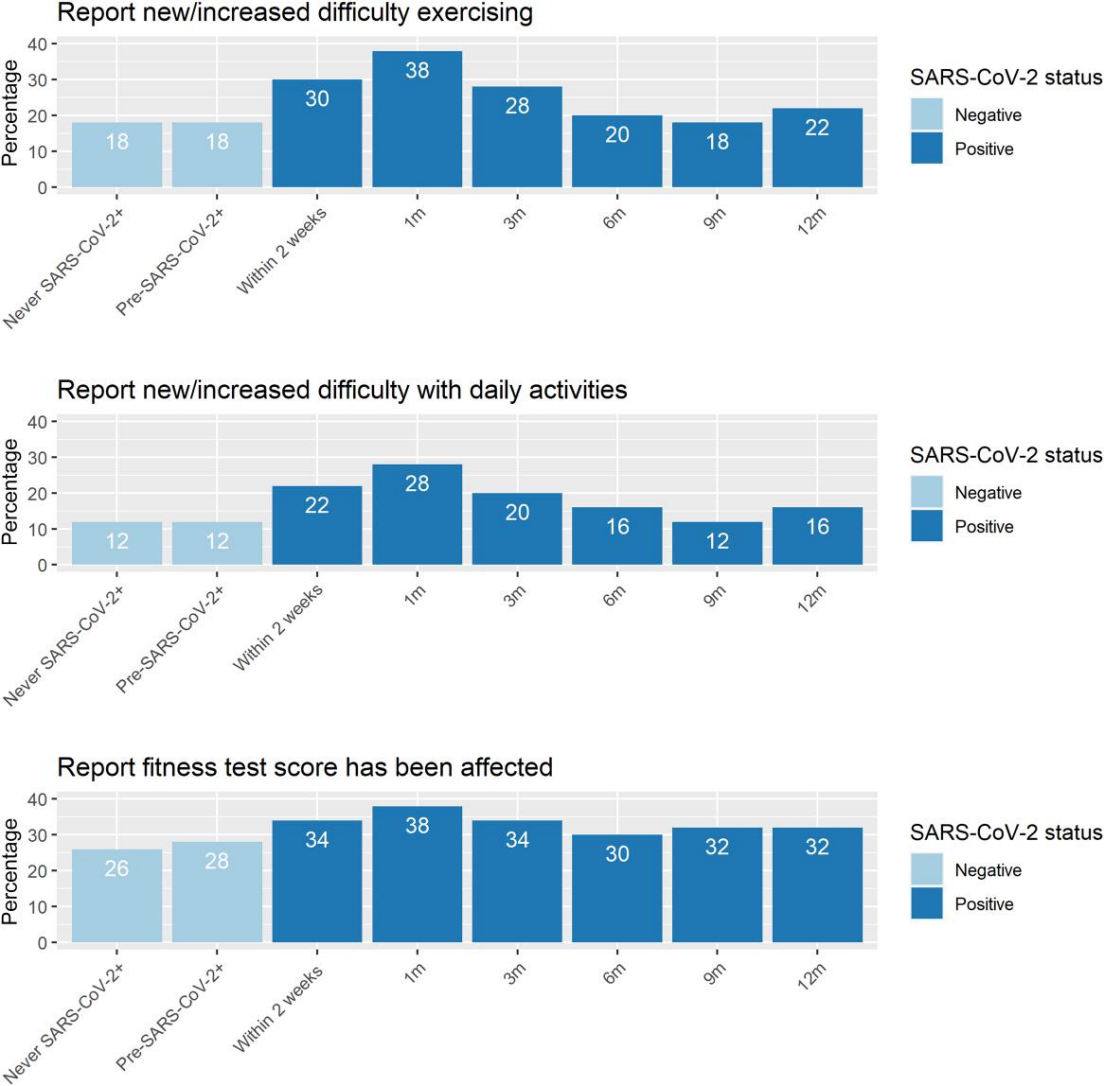
Responses to fitness survey questions, by time. The numbers in the bars represent the percentage of subjects who reported difficulty with exercise, daily activities (ie, walking or going up stairs), or physical fitness test score. Participants who never tested positive are included in the “never SARS-CoV-2+” group, and their answers are summarized over all surveys. Abbreviation: SARS-CoV-2, severe acute respiratory syndrome coronavirus 2.

More participants with a history of SARS-CoV-2 infection reported new or increased difficulty exercising and doing daily activities (ie, walking or going up stairs) on ≥1 survey than did participants without a history of SARS-CoV-2 infection (difficulty exercising 38.7% vs 18.4%; *P* < .01; difficulty with daily activities 30.4% vs 12.7%; *P* < .01) (Table 2). Among those who reported difficulties with exercise and daily activities on ≥1 survey, the most frequently cited reasons were shortness of breath or fatigue. Service members with a history of SARS-CoV-2 infection were more likely to report that their FT scores were affected (42.7% vs 26.2%; *P* < .01). The percentage reporting new or increased difficulty exercising or with daily activities was similar among those who were never SARS-CoV-2 positive and those who answered surveys before becoming SARS-CoV-2 positive (Figure 1). Among those who tested positive for SARS-CoV-2, the percentage reporting these outcomes peaked at 1 month postinfection and decreased thereafter. At 9 months postinfection, the percentage of participants reporting difficulty exercising or with daily activities was similar to that reported by those who were never positive for SARS-CoV-2. The percentage of those with a history of SARS-CoV-2 infection at 9 and 12 months postinfection who reported decreased FT score continued to be higher compared with those without a documented infection.

**Table 2.** Survey Question Responses Among Those Active Duty Participants who Responded to ≥1 EPICC Survey; Survey Responses Were Combined, and the Responses Listed Below Reflect Whether the Participants Ever Responded “Yes” to the Survey Questions if Multiple Surveys Were Completed

	SARS-CoV-2- (n = 1591), No. (%)	SARS-CoV-2+ (n = 1923), No. (%)	Total (n = 3514)	*P* Value
Do you have new/increased difficulty exercising?				<.01^a^
No	1295 (81.6)	1177 (61.3)	2472 (70.5)	
Yes	292 (18.4)	742 (38.7)	1034 (29.5)	
N/A (“I don’t exercise”)	4	4	8	
If yes, is this new/increased difficulty exercising due to any of the following (check all that apply):				
Fatigue or tiredness				.21^a^
No	78 (26.7)	171 (23.0)	249 (24.1)	
Yes	214 (73.3)	571 (77.0)	785 (75.9)	
N/A	1299	1181	2480	
Shortness of breath or difficulty breathing				<.01^a^
No	97 (33.2)	153 (20.6)	250 (24.2)	
Yes	195 (66.8)	589 (79.4)	784 (75.8)	
N/A	1299	1181	2480	
Joint pain				.40^a^
No	156 (53.4)	418 (56.3)	574 (55.5)	
Yes	136 (46.6)	324 (43.7)	460 (44.5)	
N/A	1299	1181	2480	
Difficulty moving or poor coordination				.31^a^
No	254 (87.0)	627 (84.5)	881 (85.2)	
Yes	38 (13.0)	115 (15.5)	153 (14.8)	
N/A	1299	1181	2480	
Other				.02^a^
No	261 (89.4)	694 (93.5)	955 (92.4)	
Yes	31 (10.6)	48 (6.5)	79 (7.6)	
N/A	1299	1181	2480	
Do you have new/increased difficulty doing daily activities like walking or going up stairs?				<.01^a^
No	1389 (87.3)	1339 (69.6)	2728 (77.6)	
Yes	202 (12.7)	584 (30.4)	786 (22.4)	
If yes, is this new/increased difficulty doing daily activities due to any of the following (check all that apply):				
Fatigue or tiredness				<.01^a^
No	75 (37.1)	126 (21.6)	201 (25.6)	
Yes	127 (62.9)	458 (78.4)	585 (74.4)	
N/A	1389	1339	2728	
Shortness of breath or difficulty breathing				<.03^a^
No	47 (23.3)	96 (16.4)	143 (18.2)	
Yes	155 (76.7)	488 (83.6)	643 (81.8)	
N/A	1389	1339	2728	
Joint pain				.13^a^
No	99 (49.0)	322 (55.1)	421 (53.6)	
Yes	103 (51.0)	262 (44.9)	365 (46.4)	
N/A	1389	1339	2728	
Difficulty moving or poor coordination				.55^a^
No	179 (88.6)	508 (87.0)	687 (87.4)	
Yes	23 (11.4)	76 (13.0)	99 (12.6)	
N/A	1389	1339	2728	
Other				.59^a^
No	191 (94.6)	546 (93.5)	737 (93.8)	
Yes	11 (5.4)	38 (6.5)	49 (6.2)	
N/A	1389	1339	2728	
If you are an active duty service member, do you feel like your PFT or CFT score has been affected?^a^				<.01^a^
No	1138 (73.8)	1034 (57.3)	2172 (64.9)	
Yes	405 (26.2)	769 (42.7)	1174 (35.1)	
Missing	48	120	168	
In the past month, did you go to a gym or workout studio (indoors)	1192 (74.9)	1409 (73.3)	2601 (74.0)	.27^a^

Abbreviations: CFT, combat fitness test; EPICC, Epidemiology, Immunology, and Clinical Characteristics of Emerging Infectious Diseases with Pandemic Potential; PFT, physical fitness test; SARS-CoV-2, severe acute respiratory syndrome coronavirus 2.

^a^Pearson chi-square test.

**Figure 2.**
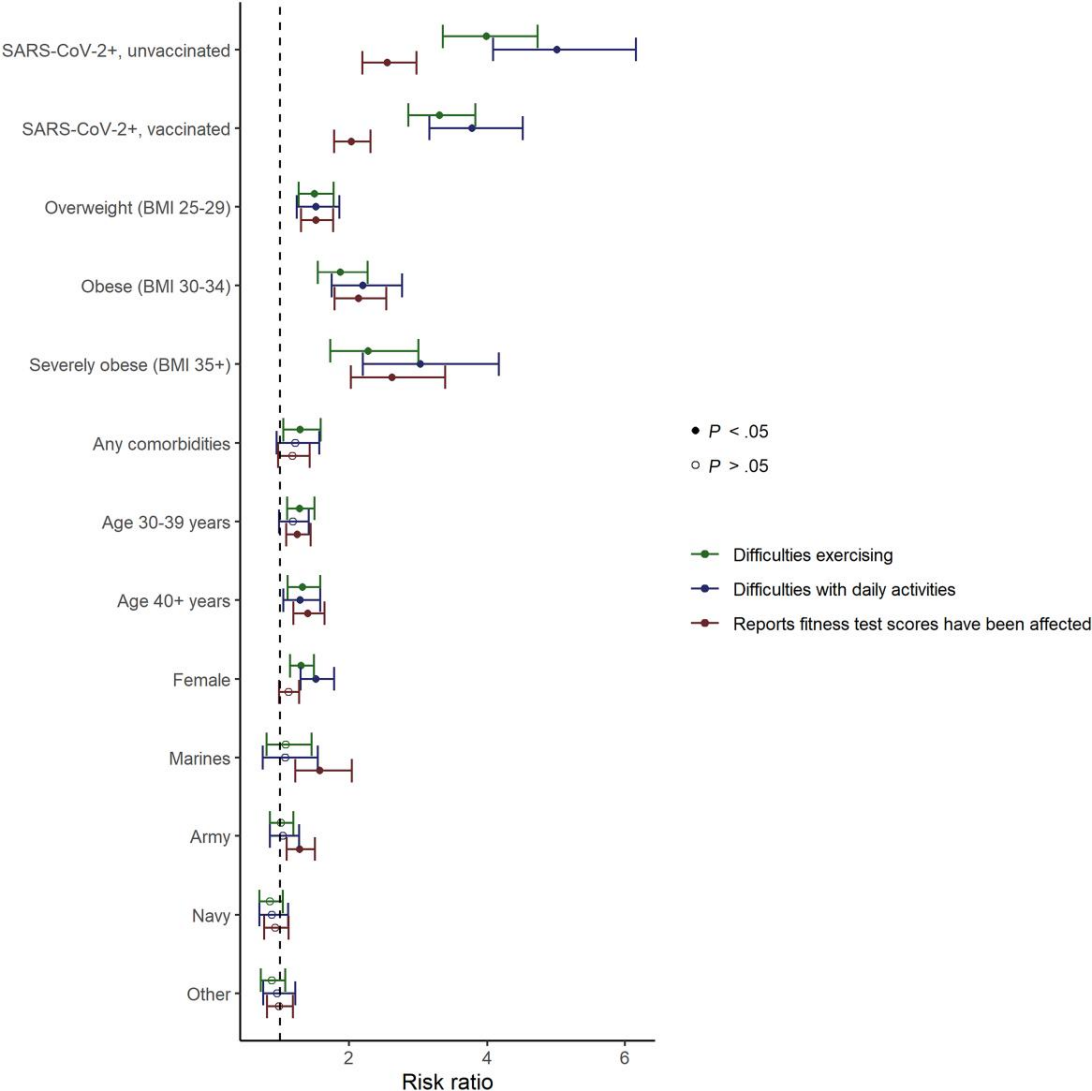
Covariates’ association with self-reported changes in fitness. Multivariable Poisson regression models fit separately for each outcome included time since first SARS-CoV-2-positive test or time since enrollment (for SARS-CoV-2-negative participants) and random effect for the participant in addition to the listed covariates. The reference category for BMI is normal/underweight; the reference category for military service branch is Air Force; the reference category for age is 18–29 years; the reference category for SARS-CoV-2 status is uninfected. Abbreviations: BMI, body mass index; SARS-CoV-2, severe acute respiratory syndrome coronavirus 2.

SARS-CoV-2 infection history was strongly associated with reports of perceived impaired fitness across all 3 outcome measures even after adjustment for BMI category, age, sex, service, time since infection/enrollment, and comorbidities (Figure 2; Supplementary Table 3). Among those with a history of SARS-CoV-2 infection, full vaccination was associated with a 19%, 24%, and 13% lower risk of post-COVID-19 difficulty exercising (adjusted risk ratio [aRR], 0.81; 95% CI, 0.70–0.95), difficulty with daily activities (aRR, 0.76; 95% CI, 0.64–0.91), and decreased FT scores (aRR, 0.87; 95% CI, 0.76–1.00), respectively (Table 3; Supplementary Table 4), when compared with those who were unvaccinated. Infection after being boosted was associated with 38%, 48%, and 41% lower risk of post-COVID-19 difficulty exercising (aRR, 0.62; 95% CI, 0.51–0.74), difficulty with daily activities (aRR, 0.52; 95% CI, 0.41–0.65), and decreased FT scores (aRR, 0.59; 95% CI, 0.49–0.70), respectively, when compared with those who were unvaccinated.

**Table 3.** Multivariable Poisson Regression Models Fit Separately for Each Outcome Among Those Participants With a History of SARS-CoV-2 Infection; Models Included Time Since First SARS-CoV-2 Positive and Random Effect for the Participant in Addition to Listed Covariates

	Reported New/Increased Difficulty With Exercise (Adjusted)	*P* Value	Reported New/Increased Difficulty With Daily Activities (Adjusted)	*P* Value	Reported Fitness Test Score Was Affected (Adjusted)	*P* Value
Vaccination status						
Unvaccinated	Ref		Ref		Ref	
Fully vaccinated	0.81 (0.70, 0.95)	.009	0.76 (0.64, 0.91)	<.01	0.87 (0.76, 1.00)	.049
Boosted	0.62 (0.51, 0.74)	<.0001	0.52 (0.41, 0.65)	<.0001	0.59 (0.49, 0.70)	<.0001
Body mass index category						
Under/normal weight (BMI <25 kg/m^2^)	Ref		Ref		Ref	
Overweight (BMI 25–29 kg/m^2^)	1.34 (1.12, 1.61)	<.01	1.44 (1.15, 1.79)	<.01	1.46 (1.23, 1.73)	<.0001
Obese (BMI 30–34 kg/m^2^)	1.68 (1.37, 2.05)	<.0001	1.95 (1.53, 2.49)	<.0001	1.98 (1.64, 2.39)	<.0001
Severely obese (BMI 35+ kg/m^2^)	1.79 (1.34, 2.38)	<.001	2.50 (1.79, 3.49)	<.0001	1.97 (1.50, 2.59)	<.0001
Any comorbidities	1.18 (0.94, 1.48)	.15	1.02 (0.77, 1.34)	.90	1.06 (0.86, 1.31)	.59
Age group						
18–29 y	Ref		Ref		Ref	
30–39 y	1.22 (1.04, 1.43)	.02	1.13 (0.93, 1.37)	.21	1.26 (1.08, 1.46)	<.01
40+ y	1.34 (1.11, 1.62)	<.01	1.44 (1.15, 1.79)	.01	1.50 (1.26, 1.78)	<.0001
Sex: female	1.31 (1.14, 1.51)	<.001	1.50 (1.27, 1.77)	<.0001	1.16 (1.02, 1.33)	.03
Service branch						
Air Force	Ref		Ref		Ref	
Marines	0.72 (0.51, 1.02)	.06	0.67 (0.43, 1.03)	.07	1.13 (0.85, 1.51)	.41
Army	0.93 (0.78, 1.10)	.38	0.94 (0.76, 1.16)	.55	1.20 (1.02, 1.42)	.03
Navy	0.83 (0.67, 1.02)	.07	0.84 (0.66, 1.07)	.16	0.93 (0.76, 1.13)	.46
Other	0.87 (0.70, 1.07)	.19	0.98 (0.76, 1.26)	.85	1.03 (0.84, 1.26)	.80

Abbreviations: BMI, body mass index; SARS-CoV-2, severe acute respiratory syndrome coronavirus 2.

## DISCUSSION

Participants with a history of SARS-CoV-2 infection were more likely to report difficulties exercising and with daily activities, as well as decreased FT scores, and these limitations persisted in some participants for 6 months. This finding was noted even after adjustment for potential confounding variables and even though most of these cases (97.5%) had a relatively mild initial COVID-19 illness. In an analysis performed in the subset of participants with a history of SARS-CoV-2 infection, vaccination and boosting were associated with lower risk of fitness impairment, and this finding was consistent across variant periods.

A substantial proportion of US service members in this cohort reported that their service-mandated FT scores were affected after COVID-19; this proportion was significantly higher than in those without a known history of SARS-CoV-2 infection. We noted that service members still reported that their FT scores were affected for ≥12 months, although this may reflect the frequency of FT testing (typically once to twice per year). While these surveys represent self-reported perceptions of fitness, these findings correlate with the recent study of Swiss military members who were found to have a lower aerobic threshold evaluated ≥6 months after SARS-CoV-2 infection compared with those without a history of SARS-CoV-2 [23, 24].

Our analyses indicated a lower risk of reporting these post-COVID-19 fitness complications following the receipt of a vaccine booster dose, indicating the value of vaccination even in those who are unlikely to have severe acute COVID-19 illness. This finding is particularly noteworthy given that only a minority of US military service members are currently boosted [19] and may inform future vaccine guidance in this and other populations. We also noted that even without boosting, those with a history of vaccination were less likely to report fitness loss–related symptoms compared with those who had not received vaccination before their COVID-19 illness. While not the primary predictor of interest, we also noted that BMI category, age, and sex remained independently associated with self-reported impaired fitness. These findings may offer a means to inform longer-term prognostication for those presenting with mild COVID-19.

This analysis has several strengths, including a large nationwide cohort with 12-month follow-up and comprehensive measurement of several confounders, which allowed for multivariable adjustment for important fitness-related factors (such as age) that differed between those with and without a history of SARS-CoV-2 infection. The timing of the enrollment (before and after implementation of mandatory COVID-19 vaccination in US military active duty populations) allowed comparison of those with and without COVID-19 vaccine receipt. The negative control group allowed us to distinguish between the prevalence of these outcomes in those without a known history of SARS-CoV-2 infection and among those who had been infected with SARS-CoV-2. This control group is important in any analysis of long COVID, as many of the symptoms and outcomes are common among uninfected people [25, 26]. It is possible that some participants in the uninfected group may have been infected but were asymptomatic and therefore did not have a history of a positive test; this potential misclassification would decrease our ability to detect a difference between the groups, and our findings may therefore underestimate the true effect size.

There were several limitations, including evolving fitness testing requirements and variable access to fitness facilities over the course of the pandemic (Supplementary Figure 2), as well as a period of interruption in administration of the service fitness tests. During the early period of the pandemic, access to gyms and group exercise was curtailed and likely decreased physical fitness for many people, even those without a documented history of SARS-CoV-2 infection, which would lower our ability to detect a difference between the groups. The inclusion of people without a documented history of SARS-CoV-2 infection and resultant comparison of time-varying trends allowed us to examine this threat to validity (Supplementary Figure 2). Indeed, we showed that this difference in reported fitness score persisted even as reports of gym use increased and maintained similar frequencies between those with and without SARS-CoV-2.

Another study limitation is that we measured fitness through self-reported surveys rather than objective FT scores themselves, as they were not available in this study. However, the correlation of 3 independent survey questions suggested construct validity of these methods of measuring fitness impairment. Nevertheless, further study using actual FT scores and objective measurement of predicted exercise capacity will be important to validate these findings. Importantly, prior research has shown a correlation between post-COVID-19 symptoms and objective cardiopulmonary exercise testing. Ladlow et al. noted that persistent cardiorespiratory symptoms after COVID-19 correlated with functional limitation on objective cardiopulmonary exercise testing [27, 28] and that a high percentage of COVID-19-exposed military-trained individuals were considered “medically nondeployable” [24], in part due to reduced endurance and impaired metabolic efficiency. These symptoms may be the result of subnormal cardiac output and peripheral factors like muscle mass and mitochondrial function [28].

When we compared service members who were not included in the analysis (Supplementary Figure 1) with those who were included, we found that those who were not included were more likely to be younger, non-White, and enrolled during the pre–Delta variant period (Supplementary Table 5). These differences are likely due to the later addition of these questions to the data collection process, meaning that participants who were infected early in the pandemic were less likely to have received or responded to a survey that included the fitness questions. The bias introduced by nonresponse to the surveys may result in an overestimate of the association between SARS-CoV-2 infection and fitness impairment, as the participants who did not respond to the surveys may be less likely to have experienced longer-term symptoms associated with COVID-19 than those who responded. However, more than half of the people excluded from the analysis were enrolled early in the study, before the surveys were implemented; therefore, we expect that their exclusion was primarily based on timing of their enrollment relative to the timing of the implementation of the surveys.

This analysis excluded non–service member MHS beneficiaries, resulting in a sample that is more generalizable to a younger and likely healthier segment of the general US population. In addition, the availability of vaccines changed during the different variant periods, which limits our ability to describe the independent effects of variant and vaccination/boosting. However, we found similar results when we ran our analyses in the different variant periods; the effect of boosting on fitness impairment in the Omicron period remained statistically significant (Supplementary Tables 6–8).

These findings prompt further study in this and similar populations, as well as those with a higher frequency of comorbidities and a range of other age groups. In particular, objective end points (such as the FT scores themselves) could be used, particularly if available as a continuous variable. Cardiorespiratory evaluation such as spirometry, chest imaging, and cardiopulmonary exercise testing could be used to understand the mechanism of these self-reported fitness symptoms (eg, occult pulmonary emboli, small airways disease, or nonspecific deconditioning and muscle weakness) [3]. Finally, peripheral blood biomarkers, such as inflammatory and immune responses or antigenemia persistence, could be studied to understand if such pathogenic processes explain part of this post-COVID phenotype, with relevance for future therapeutic studies.

Although the long-term effects of SARS-CoV-2 infection with newer variants are unclear, our analysis offers important results regarding the apparent protection from vaccination and boosting against the long-term fitness impacts of COVID-19. These results may support the planning of vaccine implementation and public health messaging among young and healthy populations, including the military.

## Supplementary Material

ofad579_Supplementary_Data

## Supplementary Data

Supplementary materials are available at *Open Forum Infectious Diseases* online. Consisting of data provided by the authors to benefit the reader, the posted materials are not copyedited and are the sole responsibility of the authors, so questions or comments should be addressed to the corresponding author.
