# Supplementary material for: Decreased Self-reported Physical Fitness Following SARS-CoV-2 Infection and the Impact of Vaccine Boosters in a Cohort Study
Source: Open Forum Infect Dis. 2023 Nov 17;10(12):ofad579. doi: 10.1093/ofid/ofad579 (PMC10733205; doi:10.1093/ofid/ofad579)

**Supplementary Materials - Decreased self-reported physical fitness following SARS-CoV-2 infection and the impact of vaccine boosters in a cohort study**

**Richard et al.**

**Supplementary Table 1. EPICC study procedures by military treatment facility (MTF) or online enrollment pathway**

|  | Day 0 | Day 7 | Day 14 | Day 28 | Month  3 | Month  6 | Month  9 | Month 12 |
| --- | --- | --- | --- | --- | --- | --- | --- | --- |
| Virology (nasal, nasopharyngeal, oral swabs) | MTF |  | MTF |  |  |  |  |  |
| Blood: clinic-collected serum | MTF | MTF | MTF | MTF |  | MTF |  | MTF |
| Blood: home self-collected Mitra kits) | MTF + Online | MTF | MTF | MTF + Online |  | MTF + Online |  | MTF + Online |
| Clinical characteristics FLU-PRO plus symptom survey | MTF – Daily for days 0-14 | | |  |  |  |  |  |
| Clinical characteristics survey | MTF + Online |  |  | MTF + Online | MTF + Online | MTF + Online | MTF + Online | MTF + Online |

**Supplementary Table 2. Survey questions included in this analysis**

| Do you have new/increased difficulty doing daily activities like walking or going up stairs? | Yes, No |
| --- | --- |
| If yes, is this new/increased difficulty doing daily activities due to any of the following (check all that apply): | Shortness of breath or difficulty breathing  Fatigue or tiredness  Difficulty moving or poor coordination  Joint pain  Other |
| Please describe other reason for difficulty doing daily activities | Text field |
| Do you have new/increased difficulty exercising? | Yes, No, I don't exercise |
| If yes, is this new/increased difficulty exercising due to any of the following (check all that apply): | Shortness of breath or difficulty breathing  Fatigue or tiredness  Difficulty moving or poor coordination  Joint pain  Other |
| Please describe other reason for difficulty doing exercise | Text field |
| If you are an active service member, do you feel like your Physical Fitness Test (PFT) or Combat Fitness Test (CFT) score has been affected? | Yes, No |
| If yes, please describe how your fitness test score has been affected. | Text field |

**Supplementary Table 3. Multivariable Poisson regression models fit separately for each outcome included time since first SARS-CoV-2 positive or time since enrollment (for SARS-CoV-2 negative participants) and random effect for the participant in addition to listed covariates. BMI = body mass index; reference category for BMI is normal/underweight; reference category for military service branch is Air Force; reference category for age is 18-29 years; reference category for SARS-CoV-2 status is uninfected.**

| **Variable** | **Reported new/increased difficulty with exercise (unadjusted)** | **p-value** | **Reported new/increased difficulty with exercise (adjusted)** | **p-value** | **Reported new/increased difficulty with daily activities (unadjusted)** | **p-value** | **Reported new/increased difficulty with daily activities (adjusted)** | **p-value** | **Reported fitness test score was affected (unadjusted)** | **p-value** | **Reported fitness test score was affected (adjusted)** | **p-value** | |
| --- | --- | --- | --- | --- | --- | --- | --- | --- | --- | --- | --- | --- | --- |
| **SARS-CoV-2+, vaccinated** | 3.40 (2.94, 3.95) | <0.0001 | 3.31 (2.86, 3.83) | <0.0001 | 3.93 (3.28, 4.70) | <0.0001 | 3.78 (3.16, 4.52) | <0.0001 | 2.11 (1.85, 2.41) | <0.0001 | 2.03 (1.78, 2.31) | <0.0001 |  |
| **SARS-CoV-2+, unvaccinated** | 4.10 (3.45, 4.87) | <0.0001 | 3.99 (3.36, 4.73) | <0.0001 | 5.30 (4.31, 6.52) | <0.0001 | 5.02 (4.09, 6.16) | <0.0001 | 2.68 (2.30, 3.14) | <0.0001 | 2.55 (2.19, 2.98) | <0.0001 |  |
| **Overweight (BMI 25-29)** | 1.57 (1.32, 1.87) | <0.0001 | 1.50 (1.27, 1.77) | <0.0001 | 1.55 (1.25, 1.91) | <0.001 | 1.52 (1.24, 1.86) | <0.001 | 1.63 (1.40, 1.91) | <0.0001 | 1.52 (1.30, 1.77) | <0.0001 |  |
| **Obese (BMI 30-34)** | 2.15 (1.76, 2.63) | <0.0001 | 1.87 (1.55, 2.27) | <0.0001 | 2.49 (1.96, 3.16) | <0.0001 | 2.20 (1.75, 2.76) | <0.0001 | 2.42 (2.02, 2.89) | <0.0001 | 2.13 (1.79, 2.54) | <0.0001 |  |
| **Severely obese (BMI 35 or higher)** | 2.71 (2.01, 3.65) | <0.0001 | 2.27 (1.72, 3.00) | <0.0001 | 3.68 (2.61, 5.18) | <0.0001 | 3.03 (2.20, 4.17) | <0.0001 | 2.98 (2.28, 3.90) | <0.0001 | 2.62 (2.03, 3.39) | <0.0001 |  |
| **Any comorbidities** | 1.38 (1.11, 1.71) | <0.01 | 1.29 (1.05, 1.59) | 0.02 | 1.33 (1.03, 1.71) | <0.01 | 1.22 (0.95, 1.57) | 0.05 | 1.35 (1.11, 1.64) | <0.01 | 1.18 (0.97, 1.43) | 0.11 |  |
| **Age 30-39 years** | 1.36 (1.16, 1.60) | <0.001 | 1.28 (1.10, 1.49) | <0.01 | 1.24 (1.02, 1.50) | 0.06 | 1.18 (0.99, 1.42) | 0.15 | 1.38 (1.19, 1.60) | <0.0001 | 1.25 (1.09, 1.44) | <0.01 |  |
| **Age 40+ years** | 1.43 (1.20, 1.71) | <0.0001 | 1.33 (1.11, 1.58) | <0.01 | 1.39 (1.13, 1.72) | <0.01 | 1.29 (1.05, 1.58) | 0.05 | 1.59 (1.35, 1.86) | <0.0001 | 1.40 (1.19, 1.64) | <0.0001 |  |
| **Sex: Female** | 1.21 (1.05, 1.40) | <0.01 | 1.30 (1.14, 1.49) | <0.0001 | 1.43 (1.21, 1.70) | <0.0001 | 1.52 (1.30, 1.78) | <0.0001 | 0.99 (0.87, 1.13) | 0.98 | 1.12 (0.99, 1.27) | 0.05 |  |
| **DOD: Marines** | 0.97 (0.70, 1.33) | 0.84 | 1.08 (0.81, 1.46) | 0.59 | 0.88 (0.59, 1.31) | 0.61 | 1.07 (0.75, 1.55) | 0.61 | 1.46 (1.11, 1.92) | <0.001 | 1.58 (1.22, 2.04) | <0.001 |  |
| **DOD: Army** | 1.02 (0.85, 1.22) | 0.86 | 1.01 (0.85, 1.19) | 0.90 | 1.04 (0.83, 1.29) | 0.74 | 1.04 (0.85, 1.28) | 0.72 | 1.34 (1.13, 1.58) | <0.001 | 1.29 (1.10, 1.51) | <0.001 |  |
| **DOD: Navy** | 0.88 (0.71, 1.09) | 0.25 | 0.85 (0.70, 1.04) | 0.14 | 0.93 (0.72, 1.20) | 0.70 | 0.88 (0.70, 1.12) | 0.38 | 1.01 (0.83, 1.22) | 0.78 | 0.93 (0.77, 1.12) | 0.60 |  |
| **DOD: Other** | 0.77 (0.62, 0.96) | 0.02 | 0.88 (0.72, 1.08) | 0.15 | 0.83 (0.64, 1.08) | 0.17 | 0.96 (0.75, 1.22) | 0.66 | 0.91 (0.75, 1.11) | 0.45 | 0.98 (0.81, 1.19) | 0.95 |  |

**Supplementary Table 4. Poisson regression models fit separately for each outcome among those participants with a history of SARS-CoV-2 infection. Models included time since first SARS-CoV-2 positive and random effect for the participant. BMI = body mass index (kg/m^2^).**

| **Variable** | **Reported new/increased difficulty with exercise (unadjusted)** | **p-value** | **Reported new/increased difficulty with daily activities (unadjusted)** | **p-value** | **Reported fitness test score was affected (unadjusted)** | **p-value** |
| --- | --- | --- | --- | --- | --- | --- |
| **Unvaccinated** | **Ref** |  | **Ref** |  | **Ref** |  |
| **Fully vaccinated** | 0.81 (0.70, 0.95) | <0.01 | 0.75 (0.62, 0.90) | <0.01 | 0.86 (0.75, 0.99) | 0.04 |
| **Boosted** | 0.64 (0.53, 0.77) | <0.0001 | 0.53 (0.42, 0.67) | <0.0001 | 0.61 (0.51, 0.73) | <0.0001 |
| **Under/normal weight (BMI<25)** | **Ref** |  | **Ref** |  | **Ref** |  |
| **Overweight (BMI 25-29)** | 1.57 (1.32, 1.87) | <0.0001 | 1.41 (1.13, 1.76) | <0.01 | 1.54 (1.30, 1.83) | <0.0001 |
| **Obese (BMI 30-34)** | 2.15 (1.76, 2.63) | <0.0001 | 2.00 (1.57, 2.54) | <0.0001 | 2.12 (1.76, 2.56) | <0.0001 |
| **Severely obese (BMI 35 or higher)** | 2.71 (2.01, 3.65) | <0.0001 | 2.59 (1.85, 3.62) | <0.0001 | 2.14 (1.62, 2.81) | <0.0001 |
| **Any comorbidities** | 1.30 (1.05, 1.62) | 0.02 | 1.21 (0.93, 1.59) | 0.16 | 1.24 (1.01, 1.53) | 0.04 |
| **Age 18-29 years** | **Ref** |  | **Ref** |  | **Ref** |  |
| **Age 30-39 years** | 1.23 (1.05, 1.44) | 0.01 | 1.12 (0.92, 1.36) | 0.25 | 1.31 (1.13, 1.53) | <0.001 |
| **Age 40+ years** | 1.35 (1.13, 1.61) | 0.00 | 1.36 (1.10, 1.68) | <0.01 | 1.53 (1.30, 1.81) | <0.0001 |
| **Sex: Female** | 1.22 (1.05, 1.40) | 0.01 | 1.38 (1.16, 1.63) | <0.001 | 1.02 (0.89, 1.18) | 0.74 |
| **DOD: Air Force** | **Ref** |  | **Ref** |  | **Ref** |  |
| **DOD: Marines** | 0.97 (0.70, 1.33) | 0.84 | 0.61 (0.39, 0.95) | 0.03 | 1.07 (0.80, 1.45) | 0.64 |
| **DOD: Army** | 1.02 (0.85, 1.22) | 0.86 | 0.95 (0.76, 1.18) | 0.61 | 1.24 (1.04, 1.48) | 0.02 |
| **DOD: Navy** | 0.88 (0.71, 1.09) | 0.25 | 0.95 (0.74, 1.22) | 0.68 | 1.03 (0.84, 1.26) | 0.79 |
| **DOD: Other** | 0.77 (0.62, 0.96) | 0.02 | 0.93 (0.72, 1.21) | 0.60 | 1.00 (0.81, 1.24) | 0.99 |

**Supplementary Table 5. Comparison of active duty participants who were and were not included in the analysis (because of incomplete demographic data, multiple SARS-CoV-2 infections, and/or missing surveys).**

|  | Not included (N=1950) | Included (N=3511) | Total (N=5461) | p value |
| --- | --- | --- | --- | --- |
| **Age (years)** |  |  |  | < 0.01^1^ |
| Median (Q1, Q3) | 30.4 (25.0, 37.9) | 34.0 (27.0, 40.0) | 33.0 (26.0, 39.0) |  |
| Min - Max | 18.0 - 65.3 | 18.0 - 65.0 | 18.0 - 65.3 |  |
| **Sex** |  |  |  | 0.99^2^ |
| Male | 1392 (71.4%) | 2508 (71.4%) | 3900 (71.4%) |  |
| Female | 557 (28.6%) | 1003 (28.6%) | 1560 (28.6%) |  |
| Missing | 1 | 0 | 1 |  |
| **Race/ethnicity** |  |  |  | < 0.01^2^ |
| Asian | 120 (6.2%) | 176 (5.0%) | 296 (5.4%) |  |
| Black | 218 (11.2%) | 259 (7.4%) | 477 (8.7%) |  |
| Hispanic or Latino | 403 (20.7%) | 534 (15.2%) | 937 (17.2%) |  |
| Missing | 28 (1.4%) | 0 (0.0%) | 28 (0.5%) |  |
| Other | 187 (9.6%) | 360 (10.3%) | 547 (10.0%) |  |
| White | 994 (51.0%) | 2182 (62.1%) | 3176 (58.2%) |  |
| **BMI category** |  |  |  | 0.81^2^ |
| Under/normal weight (BMI<25) | 377 (24.4%) | 862 (24.6%) | 1239 (24.5%) |  |
| Overweight (BMI 25-29) | 761 (49.4%) | 1770 (50.4%) | 2531 (50.1%) |  |
| Obese (BMI 30-34) | 331 (21.5%) | 714 (20.3%) | 1045 (20.7%) |  |
| Severely obese (BMI 35 or higher) | 73 (4.7%) | 165 (4.7%) | 238 (4.7%) |  |
| Missing | 408 | 0 | 408 |  |
| **Charlson comorbidity index category** |  |  |  | 0.85^2^ |
| 0 | 1792 (91.9%) | 3208 (91.4%) | 5000 (91.6%) |  |
| 1-2 | 143 (7.3%) | 280 (8.0%) | 423 (7.7%) |  |
| 3-4 | 8 (0.4%) | 13 (0.4%) | 21 (0.4%) |  |
| 5+ | 6 (0.3%) | 10 (0.3%) | 16 (0.3%) |  |
| Missing | 1 | 0 | 1 |  |
| **Infected or enrolled during different variant periods** |  |  |  | < 0.01^2^ |
| Pre-Delta | 1092 (56.0%) | 1309 (37.3%) | 2401 (44.0%) |  |
| Delta | 354 (18.2%) | 1163 (33.1%) | 1517 (27.8%) |  |
| Omicron | 504 (25.8%) | 1039 (29.6%) | 1543 (28.3%) |  |

**Supplementary Table 6. Multivariable Poisson regression models considering reporting difficulties exercising as the outcome, fit separately for each variant time period and included time since first SARS-CoV-2 positive and random effect for the participant in addition to listed covariates. BMI = body mass index.**

| **Variable** | **Exercise, pre-Delta** | **P-value, pre-Delta** | **Exercise, Delta** | **P-value, Delta** | **Exercise, Omicron** | **P-value, Omicron** |
| --- | --- | --- | --- | --- | --- | --- |
| **Unvaccinated** | Ref |  | Ref |  | N/A |  |
| **Fully vaccinated** | 0.39 (0.20, 0.76) | <0.01 | 0.79 (0.57, 1.10) | 0.16 | Ref |  |
| **Boosted** | N/A |  | 0.79 (0.44, 1.41) | 0.42 | 0.71 (0.58, 0.86) | <0.001 |
| **Under/normal weight (BMI<25)** | Ref |  | Ref |  | Ref |  |
| **Overweight (BMI 25-29)** | 2.04 (1.43, 2.91) | <0.0001 | 1.05 (0.73, 1.50) | 0.81 | 1.14 (0.88, 1.48) | 0.31 |
| **Obese (BMI 30-34)** | 2.30 (1.57, 3.37) | <0.0001 | 1.38 (0.93, 2.05) | 0.11 | 1.53 (1.14, 2.06) | <0.01 |
| **Severely obese (BMI 35 or higher)** | 2.12 (1.25, 3.58) | <0.01 | 1.38 (0.75, 2.53) | 0.30 | 1.72 (1.14, 2.60) | <0.01 |
| **Any comorbidities** | 0.83 (0.54, 1.28) | 0.39 | 1.44 (0.93, 2.23) | 0.11 | 1.25 (0.90, 1.75) | 0.18 |
| **Age 18-29 years** | Ref |  | Ref |  | Ref |  |
| **Age 30-39 years** | 1.48 (1.13, 1.95) | <0.01 | 0.98 (0.71, 1.36) | 0.92 | 1.21 (0.95, 1.54) | 0.12 |
| **Age 40+ years** | 1.63 (1.18, 2.25) | <0.01 | 1.39 (0.96, 2.02) | 0.08 | 1.14 (0.85, 1.54) | 0.38 |
| **Sex: Female** | 1.39 (1.09, 1.76) | <0.01 | 1.26 (0.94, 1.71) | 0.13 | 1.21 (0.98, 1.49) | 0.08 |
| **DOD: Air Force** |  |  |  |  |  |  |
| **DOD: Marines** | 1.02 (0.58, 1.80) | 0.93 | 0.70 (0.36, 1.37) | 0.30 | 0.66 (0.38, 1.14) | 0.14 |
| **DOD: Army** | 0.81 (0.59, 1.10) | 0.18 | 1.38 (0.95, 2.02) | 0.09 | 0.86 (0.67, 1.10) | 0.24 |
| **DOD: Navy** | 0.79 (0.56, 1.11) | 0.18 | 1.16 (0.75, 1.80) | 0.49 | 0.73 (0.53, 1.01) | 0.06 |
| **DOD: Other** | 0.89 (0.61, 1.28) | 0.52 | 1.04 (0.64, 1.68) | 0.88 | 0.81 (0.60, 1.10) | 0.18 |

**Supplementary Table 7. Multivariable Poisson regression models considering reporting difficulties with daily activities as the outcome, fit separately for each variant time period and included time since first SARS-CoV-2 positive and random effect for the participant in addition to listed covariates. BMI = body mass index.**

| **Variable** | **Activities, pre-Delta** | **P-value, pre-Delta** | **Activities, Delta** | **P-value, Delta** | **Activities, Omicron** | **P-value, Omicron** |
| --- | --- | --- | --- | --- | --- | --- |
| **Unvaccinated** | Ref |  | Ref |  | N/A |  |
| **Fully vaccinated** | 0.41 (0.19, 0.88) | 0.02 | 0.65 (0.45, 0.94) | 0.02 | Ref |  |
| **Boosted** | N/A |  | 0.85 (0.45, 1.61) | 0.62 | 0.60 (0.47, 0.77) | <0.0001 |
| **Under/normal weight (BMI<25)** | Ref |  | Ref |  | Ref |  |
| **Overweight (BMI 25-29)** | 2.06 (1.34, 3.17) | <0.01 | 1.01 (0.66, 1.54) | 0.96 | 1.33 (0.96, 1.85) | 0.09 |
| **Obese (BMI 30-34)** | 2.73 (1.73, 4.32) | <0.0001 | 1.40 (0.88, 2.22) | 0.16 | 1.89 (1.31, 2.74) | <0.001 |
| **Severely obese (BMI 35 or higher)** | 3.44 (1.92, 6.17) | <0.0001 | 1.31 (0.63, 2.73) | 0.46 | 2.72 (1.68, 4.39) | <0.0001 |
| **Any comorbidities** | 0.68 (0.41, 1.13) | 0.14 | 1.40 (0.84, 2.34) | 0.20 | 1.02 (0.66, 1.57) | 0.94 |
| **Age 18-29 years** | Ref |  | Ref |  | Ref |  |
| **Age 30-39 years** | 1.41 (1.01, 1.96) | 0.04 | 0.82 (0.56, 1.21) | 0.32 | 1.17 (0.87, 1.57) | 0.29 |
| **Age 40+ years** | 1.96 (1.35, 2.85) | <0.001 | 1.34 (0.87, 2.06) | 0.19 | 1.15 (0.80, 1.65) | 0.44 |
| **Sex: Female** | 1.54 (1.16, 2.04) | <0.01 | 1.27 (0.89, 1.80) | 0.19 | 1.49 (1.16, 1.92) | <0.01 |
| **DOD: Air Force** | Ref |  | Ref |  | Ref |  |
| **DOD: Marines** | 0.82 (0.40, 1.65) | 0.57 | 0.66 (0.29, 1.52) | 0.33 | 0.64 (0.31, 1.29) | 0.21 |
| **DOD: Army** | 0.76 (0.53, 1.10) | 0.15 | 1.51 (0.96, 2.37) | 0.08 | 0.89 (0.65, 1.22) | 0.48 |
| **DOD: Navy** | 0.68 (0.46, 1.02) | 0.06 | 1.39 (0.83, 2.32) | 0.21 | 0.74 (0.50, 1.09) | 0.13 |
| **DOD: Other** | 0.87 (0.56, 1.34) | 0.53 | 1.24 (0.71, 2.19) | 0.45 | 0.98 (0.68, 1.40) | 0.90 |

**Supplementary Table 8. Multivariable Poisson regression models considering reporting that their Fitness Test (FT) scores were affected as the outcome, fit separately for each variant time period and included time since first SARS-CoV-2 positive and random effect for the participant in addition to listed covariates. BMI = body mass index.**

| **Variable** | **FT, pre-Delta** | **P-value, pre-Delta** | **FT, Delta** | **P-value, Delta** | **FT, Omicron** | **P-value, Omicron** |
| --- | --- | --- | --- | --- | --- | --- |
| **Unvaccinated** | Ref |  | Ref |  | N/A |  |
| **Fully vaccinated** | 0.98 (0.66, 1.48) | 0.94 | 0.83 (0.62, 1.10) | 0.20 | Ref |  |
| **Boosted** | N/A |  | 0.48 (0.27, 0.85) | 0.01 | 0.67 (0.55, 0.83) | <0.001 |
| **Under/normal weight (BMI<25)** | Ref |  | Ref |  | Ref |  |
| **Overweight (BMI 25-29)** | 1.96 (1.45, 2.66) | <0.0001 | 1.49 (1.06, 2.09) | 0.02 | 1.13 (0.87, 1.47) | 0.35 |
| **Obese (BMI 30-34)** | 2.22 (1.59, 3.09) | <0.0001 | 2.02 (1.39, 2.95) | <0.001 | 1.80 (1.35, 2.42) | <0.0001 |
| **Severely obese (BMI 35 or higher)** | 2.09 (1.32, 3.30) | <0.01 | 1.95 (1.12, 3.39) | 0.02 | 1.92 (1.25, 2.95) | <0.01 |
| **Any comorbidities** | 0.94 (0.65, 1.35) | 0.72 | 1.22 (0.82, 1.81) | 0.33 | 1.05 (0.75, 1.49) | 0.76 |
| **Age 18-29 years** | Ref |  | Ref |  | Ref |  |
| **Age 30-39 years** | 1.36 (1.07, 1.73) | 0.01 | 1.09 (0.80, 1.48) | 0.58 | 1.31 (1.02, 1.68) | 0.03 |
| **Age 40+ years** | 1.69 (1.28, 2.22) | <0.001 | 1.45 (1.03, 2.05) | 0.04 | 1.45 (1.08, 1.95) | 0.01 |
| **Sex: Female** | 1.17 (0.94, 1.45) | 0.17 | 1.09 (0.82, 1.44) | 0.57 | 1.16 (0.94, 1.45) | 0.17 |
| **DOD: Air Force** | Ref |  | Ref |  | Ref |  |
| **DOD: Marines** | 1.30 (0.80, 2.11) | 0.30 | 1.05 (0.60, 1.85) | 0.86 | 1.13 (0.71, 1.80) | 0.61 |
| **DOD: Army** | 1.08 (0.81, 1.44) | 0.62 | 1.63 (1.16, 2.31) | <0.01 | 1.07 (0.83, 1.38) | 0.59 |
| **DOD: Navy** | 1.05 (0.76, 1.44) | 0.79 | 1.25 (0.84, 1.86) | 0.27 | 0.65 (0.46, 0.92) | 0.01 |
| **DOD: Other** | 1.16 (0.82, 1.62) | 0.40 | 1.24 (0.80, 1.93) | 0.33 | 0.81 (0.59, 1.11) | 0.19 |

**Supplementary Figure 1.** Flow chart of inclusion in the analyses of EPICC cohort participants.

**
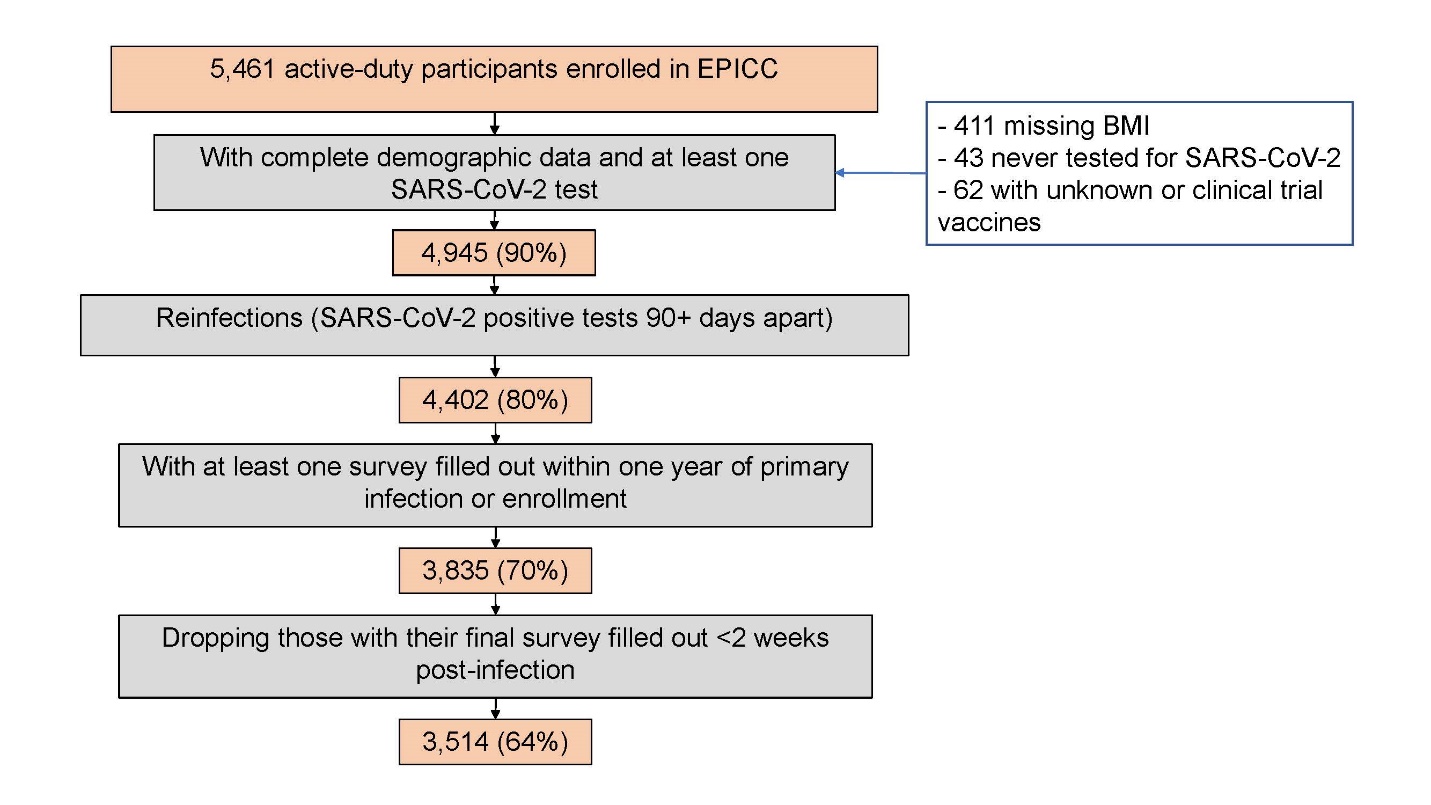
**

**Supplementary Figure 2.** **Time-varying trends of reported difficulty exercising, difficulty with daily activities, feeling like Fitness Test Scores were affected, and gym attendance (indoors) in the past month.**


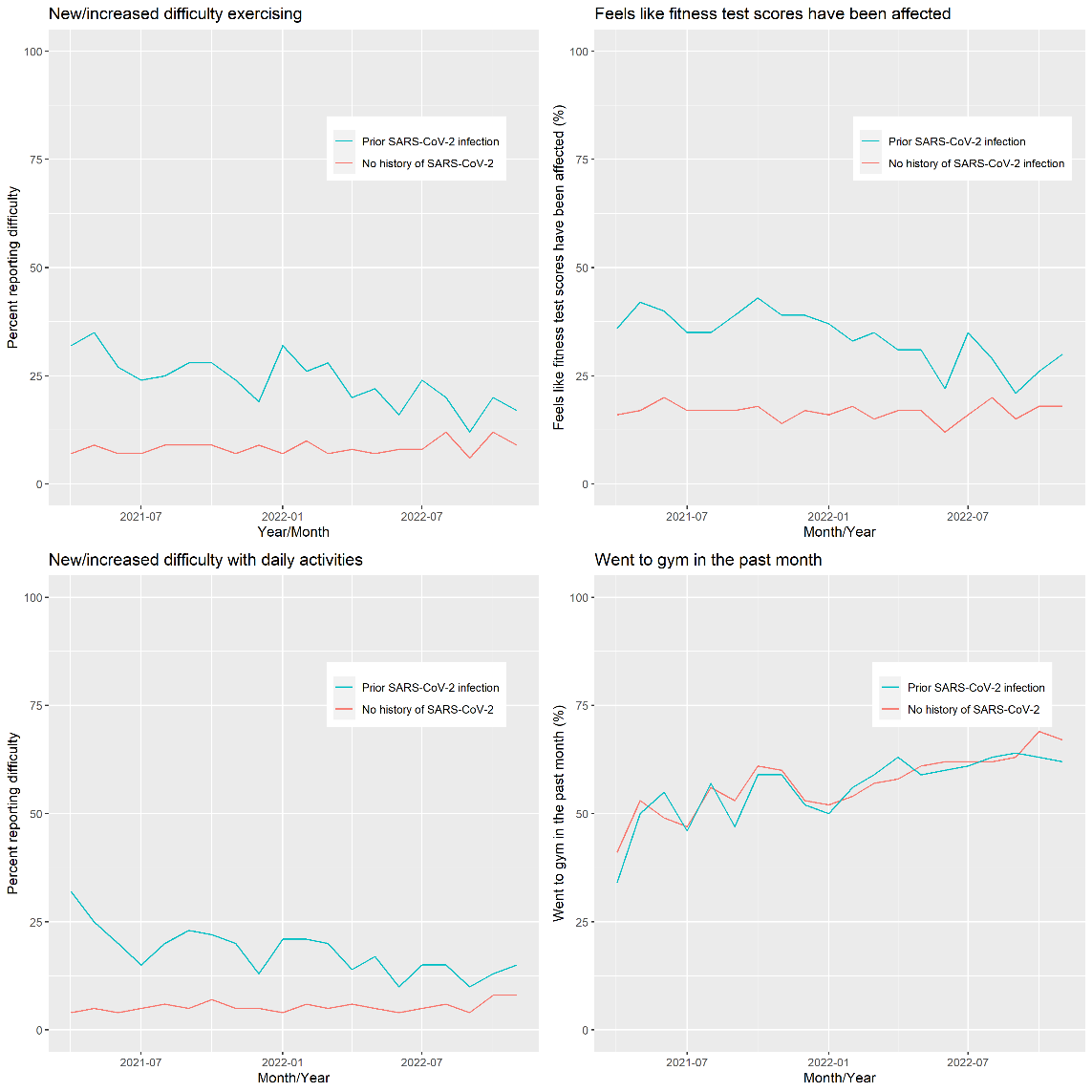

Supplement: ofad579_Supplementary_Data [file ofad579_supplementary_data.docx]
